# COVID-19 Classification on Chest X-ray Images Using Deep Learning Methods

**DOI:** 10.3390/ijerph20032035

**Published:** 2023-01-22

**Authors:** Marios Constantinou, Themis Exarchos, Aristidis G. Vrahatis, Panagiotis Vlamos

**Affiliations:** Bioinformatics and Human Electrophysiology Laboratory, Department of Informatics, Ionian University, 49132 Corfu, Greece

**Keywords:** deep learning, COVID-19, ResNet50, ResNet101, DenseNet121, DenseNet169, InceptionV3, transfer learning, chest X-rays

## Abstract

Since December 2019, the coronavirus disease has significantly affected millions of people. Given the effect this disease has on the pulmonary systems of humans, there is a need for chest radiographic imaging (CXR) for monitoring the disease and preventing further deaths. Several studies have been shown that Deep Learning models can achieve promising results for COVID-19 diagnosis towards the CXR perspective. In this study, five deep learning models were analyzed and evaluated with the aim of identifying COVID-19 from chest X-ray images. The scope of this study is to highlight the significance and potential of individual deep learning models in COVID-19 CXR images. More specifically, we utilized the ResNet50, ResNet101, DenseNet121, DenseNet169 and InceptionV3 using Transfer Learning. All models were trained and validated on the largest publicly available repository for COVID-19 CXR images. Furthermore, they were evaluated on unknown data that was not used for training or validation, authenticating their performance and clarifying their usage in a medical scenario. All models achieved satisfactory performance where ResNet101 was the superior model achieving 96% in Precision, Recall and Accuracy, respectively. Our outcomes show the potential of deep learning models on COVID-19 medical offering a promising way for the deeper understanding of COVID-19.

## 1. Introduction

In December 2019, the first case of Coronavirus 2019 (COVID019) was reported in Wuhan, China. Until now, the virus affected millions of people, showing almost 630 million cases and 6.5 million deaths worldwide [1]. The most common symptoms of COVID-19 are fever, cough, fatigue, headache, dizziness, sputum and dyspnea. Consequently, some patients sustained further damage to their respiratory system; specifically, lesions were detected in the lower lobes of both lungs. Severe cases of COVID-19 can result in acute respiratory distress syndrome or complete respiratory failure [2].

Given the solemnity of COVID-19, reliable and swift diagnosis is extremely important. There have been numerous methods for the detection of COVID-19. The primary method is reverse-transcription polymerase chain reaction (RT-PCR) [3]. These tests suffer from high false-positives or false-negatives due to sample contamination, virus mutations or user error during sample extraction [4]. As a result, several studies [5,6] suggested on using Computed Tomography (CT-Scans) for performing diagnosis, since it showed higher accuracy. Consequently, it was shown that the majority of COVID-19 cases share similar radiographic features, such as bilateral abnormalities and multifocal ground-glass opacities, mostly at the lower lung lobes during the early stages for the disease and at the final stages pulmonary consolidation was observed [7]. However, compared to CT-Scans, chest X-rays are cheaper and faster in image generation; furthermore, it is an accessible method for medical imaging and the body gets exposed to less radiation during the procedure [8]. Chest X-rays are already used as a diagnostic tool for COVID-19 [9]. Furthermore, there are some regarding the radiation exposure to patients during COVID screening. On the other hand, reducing the radiation dose lowers the image quality bringing noise and artifacts to the produced images, compromising the diagnosis. In [10], they used U-Net based discriminators in the GANs framework that enabled it to learn both global and local differences between the denoised and normal-dose images. Results based on simulated and real-world datasets showed excellent performance on denoising low-dose CT (LDCT) images, which consequently enables safer ways for patient screening. On a different study [11], they applied Neural Network Architecture Search (NAS) to LDCT and proposed a multi-scale and multi-level memory efficient NAS for LDCT denoising. Their proposed method showed better results using fewer parameters than other state-of-the-art methods.

There has been an immense growth in Machine Learning the past few years. Specifically, in medicine, it is used for various tasks, such as classification of cardiovascular diseases, diabetic retinopathy and others [12,13,14]. The revolutionary performance of the convolutional neural network (CNN), has enabled medical experts to use it on many tasks, such as the diagnosis of skin lesions, detection of brain tumors and breast cancer [15,16,17].

Applying Deep Learning models on chest X-ray (CXR) images has proven beneficial where various researchers showed auspicious results in the diagnosis of pulmonary diseases including COVID-19 pneumonia. Notably, Rajpurkar et al. [18] developed a new CNN architecture called CheXNet based on DenseNet121 for the classification of 14 different pulmonary diseases by training it on over 100,000 X-ray images. They reported that their method exceeds average radiologist performance on the F1 metric. Similarly, in [19] the authors proposed a method for automatic detection of COVID-19 pneumonia from CXR images using pre-trained convolutional neural networks, reaching accuracy ~99%. In addition, Keidar et al. [20] proposed a deep learning model for the detection of COVID-19 from CXR images and clustering of similar images to the model’s result. Lastly, in [21] a method for the detection of COVID-19 is proposed using various Deep Learning models and a support vector machine (SVM) as a classifier.

Correspondingly, additional studies proposed methods for the automatic diagnosis of COVID-19, from CXR images using Deep Learning [22,23,24,25]. Their methods revealed high performance in detecting COVID-19; although, they possess a few flaws. Foremost, all the mentioned studies had finite number of COVID-19 CXR images. This can affect the training and evaluation performance of these methods, resulting in improper generalization for future data. In addition, they did not use external unseen data for evaluation of their methods.

The goal of this study is the comparative evaluation of Deep Learning methods on COVID-19 CXR image classification and their potential to be used as decision-making tools for COVID-19 diagnosis. Our analysis is performed using five deep learning models covering various state-of-the-art architectures. We also applied all models in the largest dataset (at the time of writing and to the best of our knowledge) [26].

## 2. Materials and Methods

The COVID-QU dataset [26] is used for this study and it consists of 33,920 CXR images from three different classes. More specifically, COVID-19 contains 11,956 images of coronavirus positive patients, non-COVID-19 contains 11,263 images of viral or bacterial pneumonia patients and lastly, Normal contains 10,701 healthy images. Moreover, COVID-QU contains only posterior to anterior (PA) and anterior to posterior (AP) X-ray images. Furthermore, this dataset contains the corresponding lung masks of each image, they were not used for this study. Lastly, the COVID-QU dataset was compiled and used in [27] where the team performed infection localization and severity grading from CXR images. Then, the team decided to upload their data online making it more accessible to other researchers. The sources that were used for the compilation of this dataset are found below in detail:

### 2.1. COVID-19 CXR Dataset

This dataset consists of 11,956 COVID-19 positive X-ray images. To compile this dataset, various sources were accessed. Specifically, 10,814 images were taken from the BIMCV-COVID19+ [28] database, then 183 images were taken from a German medical school [29], 559 images were taken from SIRM [30], GitHub [31], Kaggle [32] and Eurorad [33]. Lastly, 400 images were taken from another COVID-19 repository [34].

### 2.2. RSNA CXR Dataset

This dataset consists of 8851 healthy and 6012 lung opacity X-ray images from the RSNA CXR [35] repository, where the lung opacity images belong in the non-COVID-19 class of the COVID-QU dataset.

### 2.3. Chest X-ray Pneumonia Dataset

The Chest X-ray Pneumonia [36] dataset was used to access 1300 viral pneumonia, 1700 bacterial pneumonia and 1000 healthy X-ray images. Viral and bacterial pneumonia images belong to the non-COVID-19 class of the COVID-QU dataset.

### 2.4. PadChest Dataset

From the PadChest [37] dataset, 4000 healthy and 4000 pneumonia X-ray images were used. The 4000 pneumonia images belong to the non-COVID-19 class of the COVID-QU dataset.

### 2.5. Montgomery and Shenzhen CXR Lung Masks Datasets

The Montgomery dataset [38] consists of 80 healthy and 58 tuberculosis X-ray images, along with their lung masks, and the Shenzhen dataset [39] consists of 326 normal and 336 tuberculosis X-ray images, where 566 of the total 662 images are accompanied by their lung masks.

### 2.6. QaTa-Cov19 CXR Infection Mask Dataset

The QaTa-Cov19 [40] dataset consists of almost 120,000 CXR images with their ground-truth infection masks. The researchers who created COVID-QU used these masks to train and evaluate their segmentation models that generated the rest of the segmentation masks.

Table 1 presents the distribution of data across three subsets grouped into three classes. In detail, the train subset consists of 21,715 CXR images, split into: COVID-19 with 7658 images, non-COVID-19 with 7208 images and Normal with 6849 images. Furthermore, the validation set consists of 5417 CXR images, split into: COVID-19 with 1903 images, non-COVID-19 with 1802 images and Normal with 1712 images. Lastly, the test set consists of 6788 CXR images, split into: COVID-19 with 2395 images, non-COVID-19 with 2253 images, and Normal with 2140 images.

**Figure 1:**
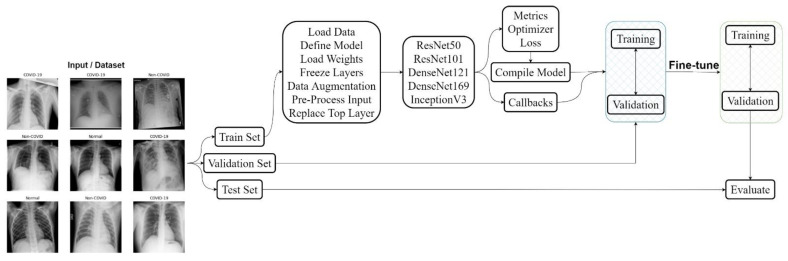
The proposed pipeline of this study.

The proposed approach for this study is demonstrated on COVID-19 classification from CXR images. In Figure 1, the general pipeline for the classification system is shown, where the first step is the configuration of the dataset into three subsets, i.e., train, validation and test sets. Step 2 consists of defining the model and all its functions where data is loaded, augmented and pre-processed and all the layers are frozen expect the classifier. Afterwards, metrics, optimizer and callbacks are defined and the model gets compiled. Step 3 consists of training only the classifier using the pre-trained weights of each model, respectively. Thereafter in step 4, fine-tuning is performed where a specific number of layers are unfrozen, and the models are trained again. Lastly, in step 5 the models are evaluated on the test set.

#### 2.6.1. Setup and Tools

The programming language that was used for the project is Python 3.10.2 in combination with Visual Studio Code version 1.69.2 as a code editor. Furthermore, regarding software version control, GitHub along with SourceTree version 3.4.9 was used. Tensorflow version 2.10 and Keras version 2.10.0 are used for the creation and training of these models. Training was performed on a personal computer with the following specs: AMD Ryzen 5600X, 16 GB RAM 3200 MHz, an RX Vega 64 and Windows 10. Since the graphics card is not compatible with Tensorflow, the training process was performed on the CPU.

##### Models and Architectures for COVID-19 Classification

Regarding COVID-19 classification, five state-of-the-art Convolutional Neural Networks (CNNs) were evaluated on COVID-19 classification from CXR images: two variants based on the ResNet [41] architecture; ResNet50 and ResNet101, then two based on the DenseNet [42] architecture; DenseNet121, and DenseNet169 and lastly, one based on the InceptionV3 [43] architecture. All models were pre-trained on the ImageNet dataset that consists of 1000 classes and millions of images.

#### 2.6.2. ResNet—Residual Network

The ResNet—Residual Network architecture [41] was proposed as a solution to the vanishing/exploding gradients problem that deep neural networks suffer. This architecture consists mostly of residual blocks and batch normalization layers, where each residual block contains convolution layers and shortcut connections.

#### 2.6.3. DenseNet

The DenseNet architecture [42], was introduced by G. Huang et al. in 2018, where each layer is connected to every other layer in a feed-forward manner. Furthermore, for each layer, the feature maps of all former layers are used as inputs and its own feature maps are used as inputs for the succeeding layers. Lastly, DenseNet solves the problem of vanishing gradients and reduces the number of parameters considerably.

#### 2.6.4. InceptionV3

InceptionV3 [43] was introduced by Szegedy et al., in 2015. The fundamental characteristic of this network is the Inception Module. This module consists of convolutions in various sizes such as 1 × 1, 3 × 3 and 5 × 5. Lastly, a pooling and concatenation layer is included.

#### 2.6.5. Image Pre-Processing

**Figure 2:**
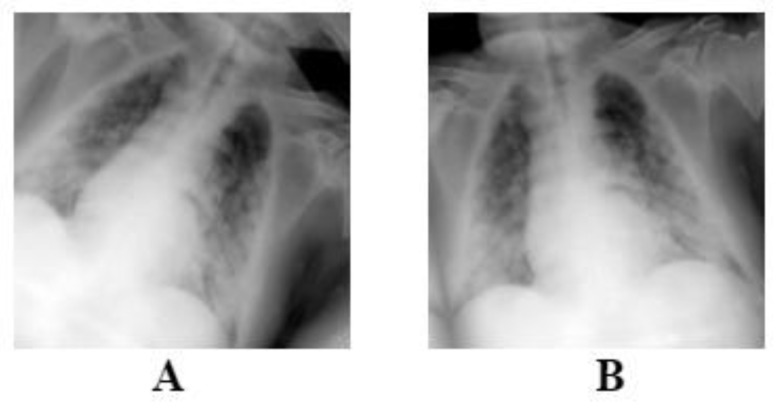
The figure above is an example of the augmentations that is performed on each image. Image (**A**) is flipped and rotated, and image (**B**) is only rotated slightly.

We utilized data augmentation methods, such as random rotation (±10°) and random horizontal flip (Figure 2), to deal with overfitting issues. The augmentations were applied randomly on each image, meaning that some images will only be rotated, flipped horizontally or both, as it is shown on Figure 2. These methods were applied on each image during model training on the training set and not before, leaving the original dataset intact without changes. Each architecture requires a specific image size; therefore, all images were resized to 224 × 224 for the ResNet and DenseNet models using bilinear interpolation. On the other hand, InceptionV3 can work with various sizes, therefore no resizing was needed.

#### 2.6.6. Model Definition

As previously mentioned, five models are trained and evaluated on CXR images. Ergo, a template was created and used for all models with only a few changes in each instance. Foremost, the base model is defined with the pre-trained weights of ImageNet and without the included classifier since a custom one is added later. Following, all layers of the base model were frozen. The model’s input is defined, then data augmentation is applied and lastly, it is pre-processed where the values of the input image are normalized to 0 and 1 or −1 and 1, depending on the architecture.

The last step is to define the new classifier. In detail, the classifier consists of 3 layers. The first one is a Global Average Pooling layer, or in the case of InceptionV3 a flatten layer, followed by a Dropout layer with a factor of 0.2, and lastly, a 3-unit Dense layer with the softmax activation function show in Equation (1) and the HeNormal kernel initializer. Regarding Equation (1), Z represents the values from the output layer and K is the number of classes / possible outcomes.

(1) $${\sigma (\vec{z})}_{i}=\frac{\exp^{z_{i}}}{\sum_{j=1}^{K}\exp^{z_{j}}}$$

#### 2.6.7. Evaluation Metrics and Callbacks

Several metrics were used to monitor the performance of each model. Specifically, Categorical Accuracy, Precision, Recall and F1-Score as shown in Equations (2)–(5), along with True Positives, True Negatives, False Positives and False Negatives. Regarding the optimization method, Adam was used with an initial learning rate of 4 × 10^−3^, 0.9 for beta 1, 0.999 for beta 2 and 1 × 10^−7^ for epsilon. Lastly, categorical cross entropy was used as a loss function as shown in Equation (6):

(2) $$\mathrm{Categorical}\;\mathrm{Accuracy}=\frac{\mathrm{TP}+\mathrm{TN}}{\mathrm{TP}+\mathrm{TN}+\mathrm{FP}+\mathrm{FN}}$$

(3) $$\mathrm{Precision}=\frac{\mathrm{TP}}{\mathrm{TP}+\mathrm{FP}}$$

(4) $$\mathrm{Recall}=\frac{\mathrm{TP}}{\mathrm{TP}+\mathrm{FN}}$$

(5) $$F1=2\frac{\mathrm{Precision}\;\times \;\mathrm{Recall}}{\mathrm{Precision}+\mathrm{Recall}}$$

(6) $$\mathrm{Loss}=-\overset{N}{\underset{i=1}{\sum }}y_{i}\times \log{\hat{y}}_{i}$$

Categorical Accuracy represents the number of correct predictions divided by the total number of predictions. Precision represents the ratio of correctly classified positive samples to the total number of classified positive samples. Recall is the ratio between the numbers of positive samples correctly classified as positive to the total number of positive samples. In this study, Recall was the primary metric.

##### Callbacks

The last task before the initial training of each model is to define all the required callbacks. In this study, the callbacks *Model Checkpoint*, *Early Stopping*, *Reduce Learning Rate on Plateau*, *Tensorboard* and *CSVLogger* were used. In detail, *model checkpoint* was setup to save only the weights of each model, *Early Stopping* was setup with an 8-epoch patience and to restore the model’s best weights. Afterwards, *Reduce Learning Rate on Plateau* was setup to reduce the learning rate by a factor of 0.2, as shown in Equation (7), with a 3-epoch patience.

(7) new lr=initial_lr×factor

Regarding model visualization, *Tensorboard* was used to monitor the training performance of each model.

#### 2.6.8. Model Training and Fine-Tuning

After every function, parameter and callback has been setup, the initial training can commence where all the layers are frozen expect the classifier. All models were set to be trained for 100 epochs. Consequently, none of them were trained for 100 epochs, because the callback Early Stopping ends their training if no improvement in performance is observed. Following the initial model training, the fine-tuning phase takes place where some layers of each model are unfrozen and are trained again. Table 2 shows in detail the number of parameters of each model after layer unfreezing.

Once the layers are unfrozen, the model is trained for around 10–15 epochs with the same callbacks, loss function and metrics. The only difference is in the optimizer function; although Adam was used during fine-tuning, the learning was set to 4 × 10^−4^.

## 3. Results

In this chapter, the training and evaluation performance is demonstrated and compared across all models. The following tables show the metrics that were discussed above with the addition of the Support column where it shows the number of samples for each class. It can be observed that all three classes had a similar number of samples, therefore eliminating the problem of class imbalance.

### 3.1. ResNet50

Table 3 shows that ResNet50 managed to achieve 97% Precision, Recall and F1-Score regarding class COVID-19. Although, its performance drops significantly for the classes non-COVID-19 and Normal. Overall, its Recall reached 95%.

**Figure 3:**
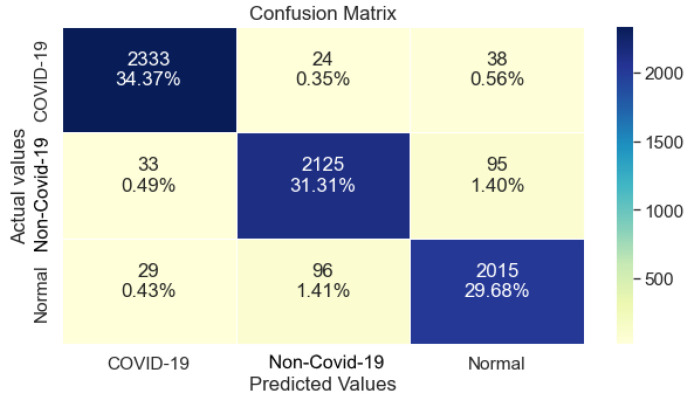
ResNet50 Confusion Matrix.

Furthermore, regarding class COVID-19, ResNet50 performed exceptionally well, as shown in Figure 3. Although, its performance degraded regarding the other two classes, with a similar number of errors.

### 3.2. ResNet101

Furthermore, ResNet101 as shown in Table 4, managed 99% Precision, 96% Recall, and 98% F1- Score regarding class COVID-19. Similar to ResNet50, a drop in performance is observed regarding the classes non-COVID-19 and Normal. Lastly, it reached 96% in Recall.

**Figure 4:**
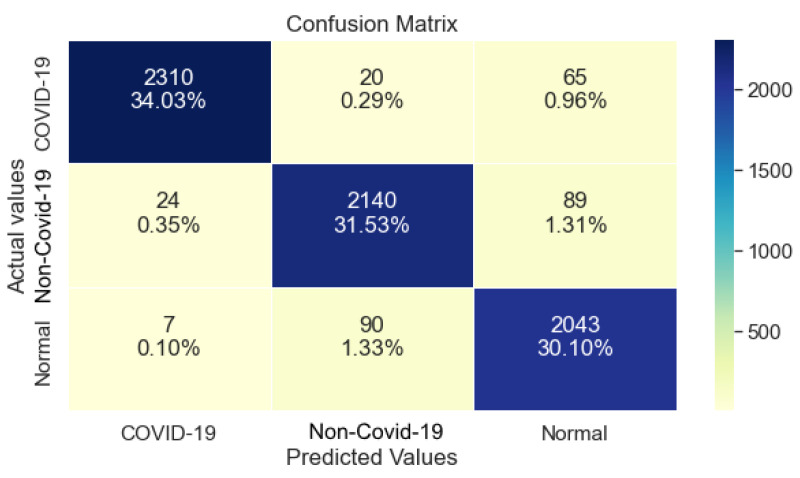
ResNet101 Confusion Matrix.

Regarding the Confusion Matrix that is shown in Figure 4, it is clear that compared to ResNet50, ResNet101 performed equally well on class COVID-19, while it also maintaining a balanced performance regarding the classes non-COVID-19 and Normal.

### 3.3. DenseNet121

DenseNet121, as shown in Table 5, managed to achieve 99% Precision, 94% Recall and 96% F1-Score regarding the class COVID-19. Furthermore, a significant drop in Precision and Recall is observed for class non-COVID-19 and Normal where it achieved 86% and 87%, respectively. The achieved Recall for this model is 93%.

**Figure 5:**
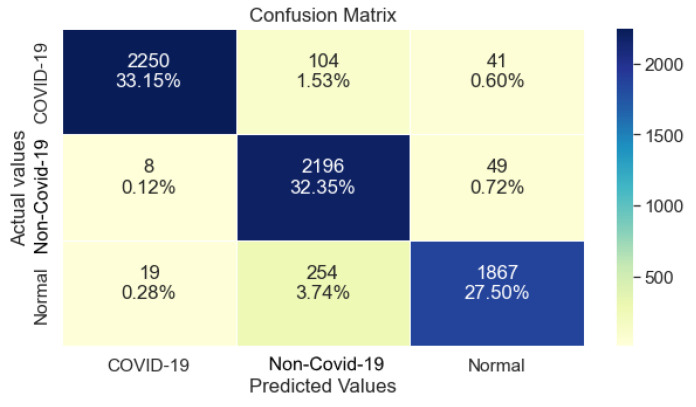
DenseNet121 Confusion Matrix.

The confusion matrix shown in Figure 5, DenseNet121 made many misclassifications regarding the classes Normal and COVID-19, where the model’s prediction classified images as non-COVID-19 in both cases. With reference to Table 5, this drop in performance is also shown by the significant drop of Precision and Recall in the classes COVID-19 and Normal, respectively.

### 3.4. DenseNet169

DenseNet169 as shown in Table 6, had similar performance with DenseNet121 despite having a larger computational capacity. Regarding the class COVID-19, 99% Precision, 93% Recall and 96% F1-Score were reported. Compared to DenseNet121, it managed to surpass its performance regarding the class non-COVID-19, but had a significant drop in its Precision regarding the class Normal. Overall, its Accuracy reached 94%.

**Figure 6:**
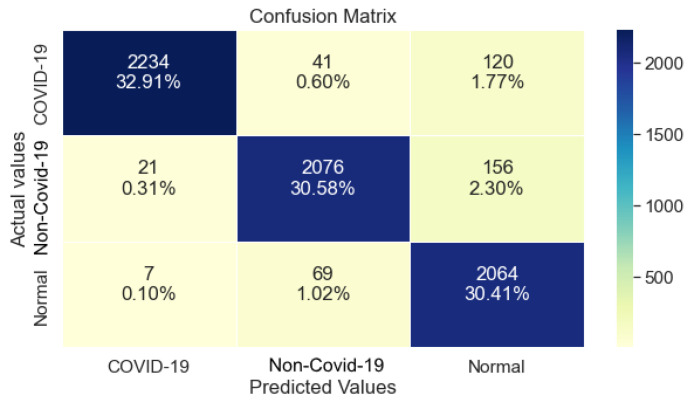
DenseNet169 Confusion matrix.

Concerning the Confusion Matrix of DenseNet169 showed in Figure 6, it is evident that misclassifications were made regarding the classes non-COVID-19 and COVID-19, where it classified images as Normal although the correct class was either COVID-19 or non-COVID-19.

### 3.5. InceptionV3

InceptionV3 as shown in Table 7, managed 97% Precision, 97% Recall and 97% F1-Score regarding the class COVID-19. Its performance on non-COVID-19 and Normal is slightly lower but balanced across all metrics. The overall Accuracy of this model is 95%.

**Figure 7:**
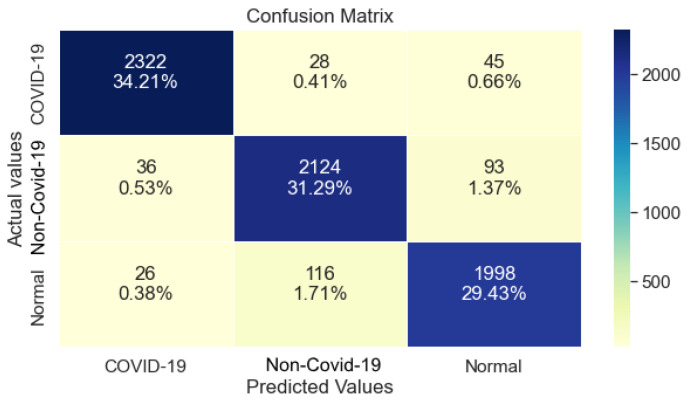
InceptionV3 Confusion Matrix.

With reference to the Confusion Matrix of this model showed in Figure 7, its performance was low regarding the class Normal, where it classified a significant number of images as non-COVID-19. Similarly, the class non-COVID-19 is troublesome, where many images were classified as Normal. Concerning the class COVID-19, it performed adequately with minimal error.

### 3.6. Overall Performance

In this study, the key metric for the classification is Recall, on the grounds that the identification of COVID-19 positive images is important, hence the requirement for high Recall on each model. All models reached high Recall values (>93%), where the top performer was ResNet101 with 96% score on all metrics as shown on Table 4 and Table 8; notwithstanding, it had the largest number of trainable parameters which translates to a larger computational capacity compared to the other 4 models.

## 4. Discussion

It is beyond doubt that COVID-19 affected millions of humans worldwide jeopardizing their health, while at the same time pushing health care services to their limit. Fast and accurate identification of positive COVID-19 cases is essential for the prevention of virus spread. CXR imaging is publicly available at a low cost while producing fast results compared to the more commonly used methods, such as RT-PCR tests and CT scans. Furthermore, LDCT scans can be used for patient screening since recent methods have been developed that successfully denoise the produced images.

Thus, numerous studies on COVID-19 identification from CXR images using deep learning methods showed excellent results. However, some of them used limited data for training and evaluation. Consequently, a model will probably not be able to generalize well to new, unseen data with insubstantial training making its usage in a clinical scenario deficient. In this study, a system is proposed for the automatic detection and diagnosis of COVID-19 from CXR images using deep learning methods. To achieve this, the largest COVID-19 CXR dataset with COVID-19 images was used to train and evaluate five different deep learning models on COVID-19 identification.

The proposed methods of this study showed high results in COVID-19 identification as shown in Table 8, attaining equal or more of 93% in Precision and Recall scores. The best performer was ResNet101, achieving 96% scores across all metrics.

Henceforth, the plan for this study is to apply lung segmentation and localization on CXR images to increase the classification accuracy of this system and also testing an ensemble model, making it more robust and enabling it to generalize even better to new CXR images. Furthermore, another goal is to test the system against professional radiologists and see how well it performs. Furthermore, collaborating with professional radiologists will also result on the acquisition of valuable feedback from them, regarding the usability of this system in a clinical environment as a decision-making tool.

It is worth mentioning that ensemble models can be a powerful tool for improving the performance of deep learning algorithms [44]. However, the scope of our work was to highlight the significance and potential of individual deep learning models, rather than to focus specifically on ensemble techniques. Therefore, we decided to evaluate each model separately and to present their results in a comparable manner. We believe that this approach allows us to gain a better understanding of the strengths and limitations of each model and to provide insights into their potential for improving the accuracy and efficiency of COVID-19 CXR image analysis.

## 5. Conclusions

In this study we evaluated five different Deep Learning models by training them on a large dataset containing CXR images of lungs with COVID-19, other pulmonary diseases or no disease at all. Our goal was to explore the potential of various Deep Learning methods in COVID-19 identification. Our findings showed promising results where all models achieved 93% and above in recall where the best performer was ResNet101 with 96% recall score. All individual models performed adequately, which means implementing more complex methods and enhancing their learning capacity could prove even more beneficial. Henceforth, the plan for this study is to apply lung segmentation and localization on CXR images to increase the classification accuracy of this system and also to test an ensemble model, making it more robust and enabling it to generalize even better to new CXR images. Furthermore, another goal is to test the system against professional radiologists and see how well it performs. Furthermore, collaborating with professional radiologists will also result on the acquisition of valuable feedback from them, regarding the usability of this system in a clinical environment as a decision-making tool.

## Figures and Tables

**Table 1:** Overall distribution of the data used in this study.

#	Subset	COVID-19	Non-COVID-19	Normal	Total
1	Train	7658—35%	7208—33%	6849—32%	21,715—64%
2	Validation	1903—35%	1802—33%	1712—32%	5417—16%
3	Test	2395—35%	2253—33%	2140—32%	6788—20%

**Table 2:** The total parameters of each model along with the trainable and non-trainable parameters after unfreezing some layers.

Parameters	ResNet50	ResNet101	DenseNet121	DenseNet169	InceptionV3
Total	23,564,800	42,632,707	7,040,579	12,647,875	22,023,971
Trainable	14,970,880	25,040,899	5,527,299	11,059,843	17,588,163
Non-Trainable	8,593,920	17,591,80	1,513,280	1,588,032	4,435,808

**Table 3:** ResNet50 Evaluation results.

	Precision	Recall	F1-Score	Support
COVID-19	0.97	0.97	0.97	2395
Non-COVID-19	0.95	0.94	0.94	2253
Normal	0.94	0.94	0.94	2140
Accuracy			0.95	6788
Macro avg	0.95	0.95	0.95	6788
Weighted avg	0.95	0.95	0.95	6788

**Table 4:** Resnet101 Evaluation results.

	Precision	Recall	F1-Score	Support
COVID-19	0.99	0.96	0.98	2395
Non-COVID-19	0.95	0.95	0.95	2253
Normal	0.93	0.95	0.94	2140
Accuracy			0.96	6788
Macro avg	0.96	0.96	0.96	6788
Weighted avg	0.96	0.96	0.96	6788

**Table 5:** DenseNet121 Evaluation results.

	Precision	Recall	F1-Score	Support
COVID-19	0.99	0.94	0.96	2395
Non-COVID-19	0.86	0.97	0.91	2253
Normal	0.95	0.87	0.91	2140
Accuracy			0.93	6788
Macro avg	0.93	0.93	0.93	6788
Weighted avg	0.93	0.93	0.93	6788

**Table 6:** DenseNet169 Evaluation results.

	Precision	Recall	F1-Score	Support
COVID-19	0.99	0.93	0.96	2395
Non-COVID-19	0.95	0.92	0.94	2253
Normal	0.88	0.96	0.92	2140
Accuracy			0.94	6788
Macro avg	0.94	0.94	0.94	6788
Weighted avg	0.94	0.94	0.94	6788

**Table 7:** InceptionV3 Evaluation results.

	Precision	Recall	F1-Score	Support
COVID-19	0.97	0.97	0.97	2395
Non-COVID-19	0.94	0.94	0.94	2253
Normal	0.94	0.93	0.93	2140
Accuracy			0.95	6788
Macro avg	0.95	0.95	0.95	6788
Weighted avg	0.95	0.95	0.95	6788

**Table 8:** Compiled Results from all models.

Model	Accuracy	Precision	Recall	Trainable Parameters
ResNet50	95%	95%	95%	14,970,880
***ResNet101***	**96%**	**96%**	**96%**	**25,040,899**
DenseNet121	93%	93%	93%	5,527,299
DenseNet169	94%	94%	94%	11,059,843
InceptionV3	95%	95%	95%	17,588,163

## Data Availability

https://www.kaggle.com/datasets/anasmohammedtahir/covidqu (accessed on 25 August 2022).
